# Supplementary material for: Reference Values to Assess Hemodilution and Warn of Potential False-Negative Minimal Residual Disease Results in Myeloma
Source: Cancers (Basel). 2021 Sep 30;13(19):4924. doi: 10.3390/cancers13194924 (PMC8508317; doi:10.3390/cancers13194924)

Supplemental Figure S1. Bivariate flow cytometry dot plots illustrating the performance of the first 8-color antibody combination of the Euroflow approach for minimal residual disease assessment in myeloma to enumerate mast cells (CD117bright, CD45dim; in red), nucleated red blood cells (CD45-, CD38-, CD117-/+, SSClo; in yellow), and B-cell precursors (CD19+, CD45dim, CD38bright, CD81bright and CD27-; in blue). Other leukocyte populations are depicted as grey dots.

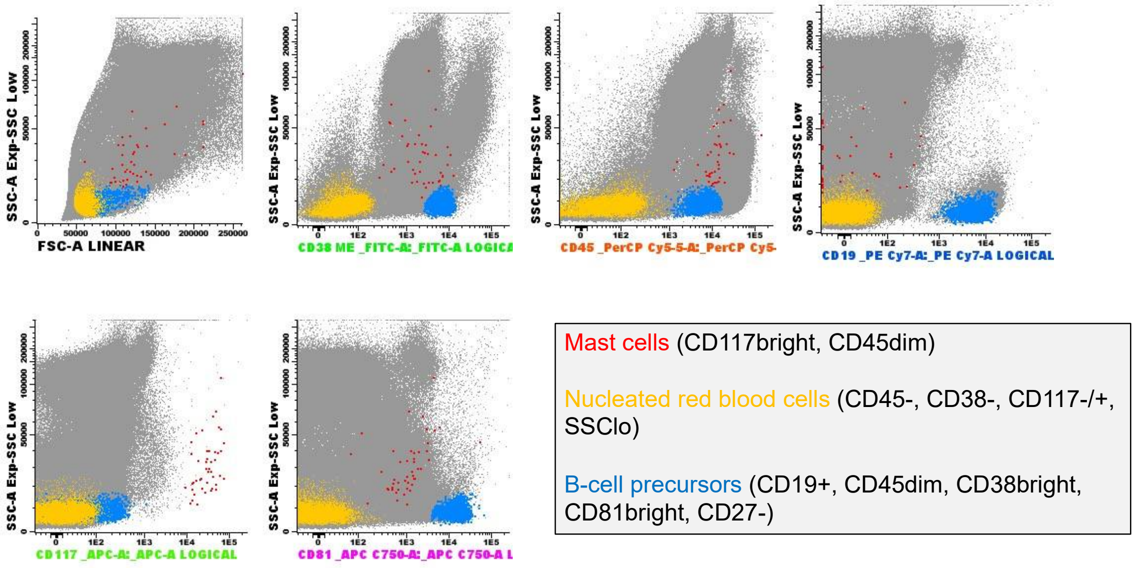

Supplement: Supplementary file 1 [file cancers-13-04924-s001.zip › cancers-1357985-supplementary.pdf]
